# *Elaphostrongylus* and *Dictyocaulus* infections in Norwegian wild reindeer and red deer populations in relation to summer pasture altitude and climate

**DOI:** 10.1016/j.ijppaw.2019.09.003

**Published:** 2019-09-11

**Authors:** Kjell Handeland, Rebecca K. Davidson, Hildegunn Viljugrein, Anders Mossing, Erling L. Meisingset, Marianne Heum, Olav Strand, Ketil Isaksen

**Affiliations:** a Norwegian Veterinary Institute, Oslo, Norway; b Norwegian Wild Reindeer Centre, Skinnarbu, Norway; c Department of Forestry and Forestry Resources, Norwegian Institute of Bioeconomy Research, Tingvoll, Norway; d Norwegian Institute for Nature Research, Trondheim, Norway; e Norwegian Meteorological Institute, Oslo, Norway

**Keywords:** Elaphostrongylus, Dictyocaulus, Deer, Cervids, Climate, Ecology

## Abstract

Nematodes of the genera *Elaphostrongylus* and *Dictyocaulus* are associated with disease in semi-domesticated tundra reindeer and farmed red deer whereas less knowledge exists in the wild. Their first stage larvae (L1) develop to the infective third stage (L3) in the environment; *Elaphostrongylus* spp. within intermediate gastropod hosts and *Dictyocaulus* spp. as free-living larvae. Larval development of *Elaphostrongylus* is highly temperature dependent with a developmental minimum of 9–10 °C. Larval development of *Dictyocaulus* spp. may occur at low temperatures (5 °C) but the larvae are sensitive to desiccation. We examined the prevalence and intensity of *Elaphostrongylus* spp. and *Dictyocaulus* spp. infections in six wild reindeer and two wild red deer populations in relation to altitude, temperature and rainfall in their respective main summer pasture area over the 5 summers prior to sampling. The parasitological examination was based upon morphological identification of L1 in the faeces of hunted animals. Altitude was calculated from animal position data and temperature and precipitation by means of a nationwide gridded data set. Temperature decreased with increasing altitude, from 13.3 °C for the lowest located red deer population (300 m) to 6.1 °C for the highest located reindeer population (1400 m). No significant relationship between altitude and rainfall was identified. *Elaphostrongylus* spp. infection decreased in prevalence with increasing altitude, being identified in 89% of investigated samples from the lowest located population and in 3% of samples from the highest. The prevalence of *Dictyocaulus* spp. infection varied between 28 and 80% and no relationship with altitude was found. The intensity of *Elaphostrongylus* spp. infection was low in reindeer and moderate in red deer whereas the intensity of *Dictyocaulus* spp. infection was moderate in both species. Our results indicated that the climatic conditions in all areas studied were suitable for *Dictyocaulus* spp., whereas summer temperature was a restrictive factor for *Elaphostrongylus* sp. in reindeer.

## Highlights

• 6 wild reindeer and 2 red deer populations investigated for nematode larvae.
• *Elaphostrongylus* spp. and *Dictyocaulus* spp. prevalence varied: 3–89% and 28–80%.
• *Elaphostrongylus* spp. prevalence decreased with increasing grazing altitude.
• No relationship between altitude and *Dictyocaulus* spp. prevalence.
• Summer temperature restrictive factor for *Elaphostrongylus* sp. in reindeer.

## Graphical abstract

**Figure:**
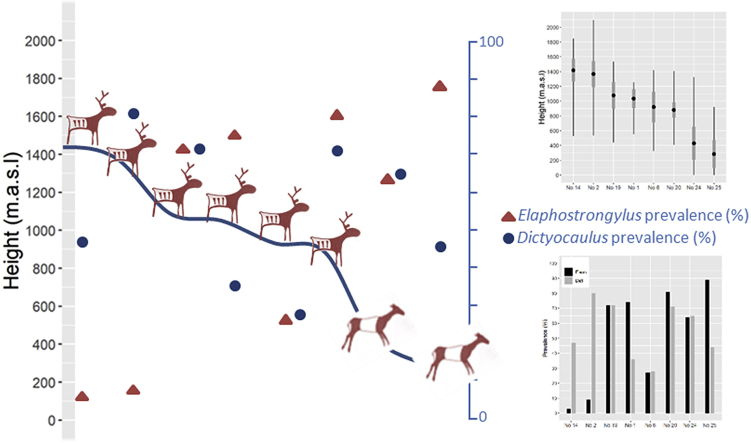

## 1 Introduction

Nematodes of the genus *Elaphostrongylus* (brain worm) are parasites and a cause of neurological disease in cervids. Reindeer (*Rangifer tarandus*) are hosts for *E. rangiferi* and red deer (*Cervus elaphus*) for *E. cervi* (Gibbons et al., 1991). *Dictyocaulus* nematodes (lungworm) infect both cervids and domestic ruminants and cause bronchitis. *Dictyocaulus* spp. found in the lungs of cervids were previously often identified as *D. viviparus* (bovine lung worm) but according to recent studies, the valid names of *Dictyocaulus* in reindeer and red deer are *D. eckerti* and *D. cervi* respectively (see Pyziel et al., 2017). Both *Elaphostrongylus* spp. and *Dictyocaulus* spp. have a climate-related ecology. This is linked to the ambient temperature and moisture dependent development of first stage larvae (L1) shed in the faeces of an infected host, to infective third stage larvae (L3) in the environment.

Larval development from L1 to L3 of *E. rangiferi* and *E. cervi* takes place in various intermediate gastropod hosts (Mitskevich, 1964; Panin, 1994; Prosl and Kutzer, 1980; Skorping and Halvorsen, 1980) and is highly temperature-dependent. Halvorsen and Skorping (1982) reported a developmental time in gastropods of L3 (*E. rangiferi*) of about 12 days at 24 °C and 2 months at 12 °C, with a developmental minimum of 9–10 °C. Hosts are infected following ingestion of gastropods containing L3 larvae that have developed during summer (June–August) (Halvorsen et al., 1980). The L3 develop to adult nematodes in the brain and spinal cord (CNS) and subsequently migrate to the skeletal muscles (Hemmingsen et al., 1993; Handeland, 1994; Handeland et al., 2000). Clinical elaphostrongylosis (neurological signs) is associated with the developmental phase in the CNS (Handeland, 1994; Handeland et al., 1994). The pre-patent period is 3–5 months (Mitskevich, 1964; Panin, 1964) and patency of infection is several years (Halvorsen et al., 1985). Due to the long pre-patent period, infected calves will normally not excrete *Elaphostrongylus* L1 larvae until after the autumn hunt. Thus infections found in hunted yearlings were generally acquired the previous year (as calves). Infections in adult animals may have occurred several years previously.

Due to the high temperature requirement for larval development of *Elaphostrongylus* spp. in gastropods, low summer temperature is considered a restrictive factor for host infection at high latitudes e.g. for semi-domesticated tundra reindeer (*Rangifer tarandus tarandus*) in northern Norway (Halvorsen et al., 1980; Halvorsen and Skorping, 1982). In this area, epizootics of clinical elaphostrongylosis in reindeer have occurred after particularly hot summers (Handeland and Slettbakk, 1994). A close association between summer temperature and the level of *Elaphostrongylus* infection could also be expected for the wild tundra reindeer populations in southern Norway, and possibly in wild red deer. Wild reindeer populations live in alpine and sub-alpine mountain areas with short and cool summers (Punsvik and Frøstrup, 2016) whereas wild red deer usually have winter grounds at lower altitudes and a majority of the populations migrate to higher inland summer pastures during spring (Mysterud et al., 2011; Bischof et al., 2012). The level of *Elaphostrongylus* infection in areas with previously marginal summer temperatures may increase as a result of climate warming. Virtually no information exists about *Elaphostrongylus* infection in Norwegian wild reindeer populations while two studies have documented the presence of this parasite in wild red deer (Ottestad, 1983; Bakka et al., 2006).

Unlike *Elaphostrongylus* spp., development of *Dictyocaulus* L1 to L3 is direct and can take place at low temperatures (5 °C) (Rose, 1956). However, the larvae are sensitive to desiccation and sufficient moisture (precipitation) is important for larval survival and host infection. Hosts are infected following ingestion of *Dictyocaulus* L3 present on the vegetation. The pre-patent period and patency of infection (*D. viviparus*) are around one and two months respectively (Taylor et al., 2007). While clinical outbreaks of dictyocaulosis have been registered in semi-domesticated reindeer herds in northern Norway (Kummeneje, 1977) no knowledge exists of this infection in wild reindeer populations. *Dictyocaulus* sp. has been recognized as the single most important nematode parasite in farmed red deer in Norway (unpublished data) and other countries, including the UK and New-Zealand (Charleston, 1980; Corrigall et al., 1980; Fletcher, 1982; Mason, 1995). Although commonly found in the lungs of autopsied wild red deer in this country, no systematic prevalence studies on *Dictyocaulus* infection have been carried out.

In the present study, we examined the prevalence and intensity of faecal *Elaphostrongylus* and *Dictyocaulus* L1 excretion in six wild reindeer and two red deer populations sampled during autumn hunting in southern Norway. The results were analyzed in relation to average altitude of each population's main summer pasture, and the calculated temperature and rainfall in this area during the previous five summers prior to sampling. Climatic calculations were extended to assess trends during the last 60 years, to look for changes in the face of climate change.

## 2 Materials and methods

### 2.1 Populations and summer pasture altitudes

**Fig. 1:**
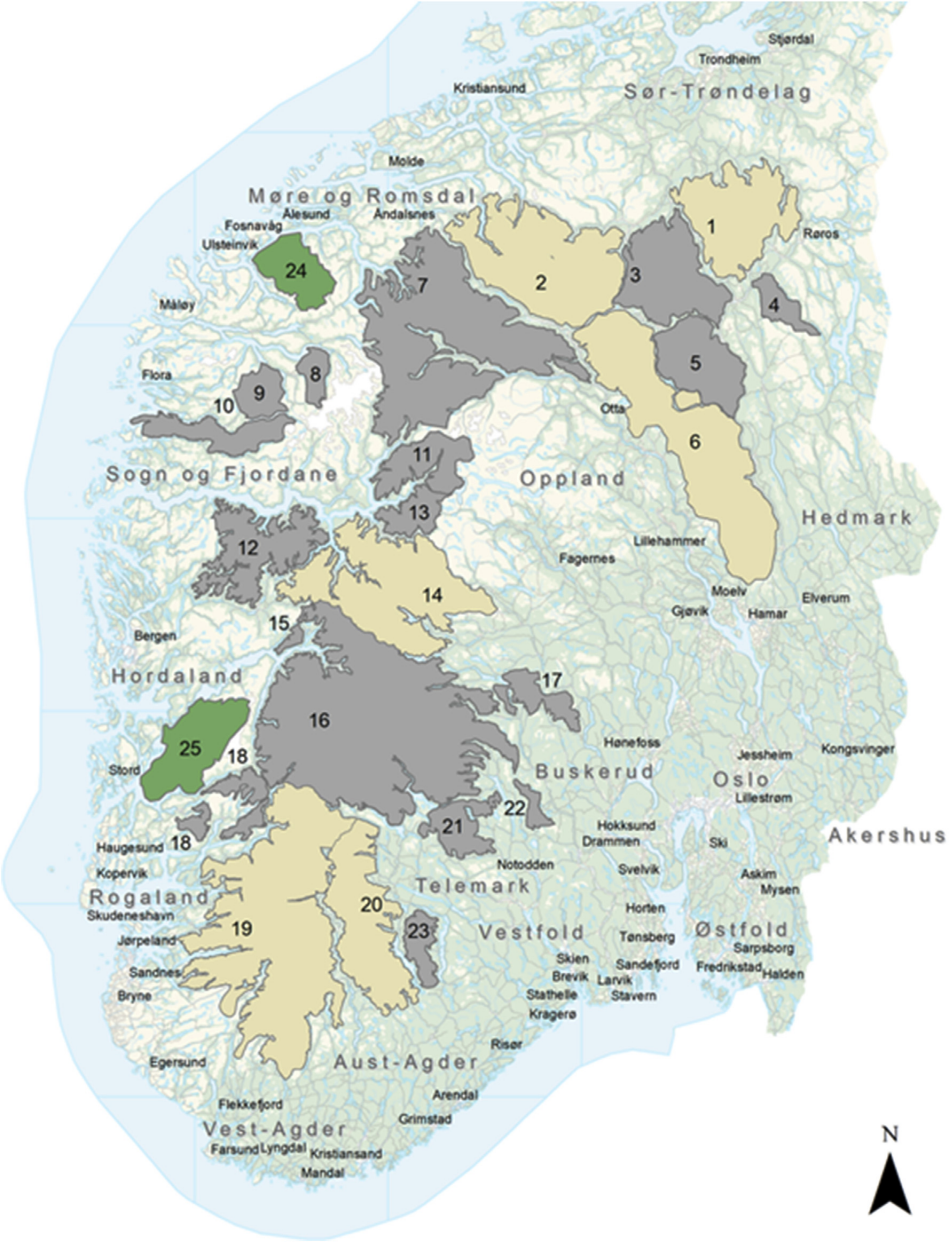
Map of South Norway showing the location of the 23 Norwegian wild tundra reindeer populations (No 1–23). The six populations included in the present study (No 1, 2, 6, 14, 19, 20) are marked with brighter tan. The location of two wild red deer municipalities studied (No 24, 25) are marked in green. (For interpretation of the references to color in this figure legend, the reader is referred to the Web version of this article.)

**Fig. 2:**
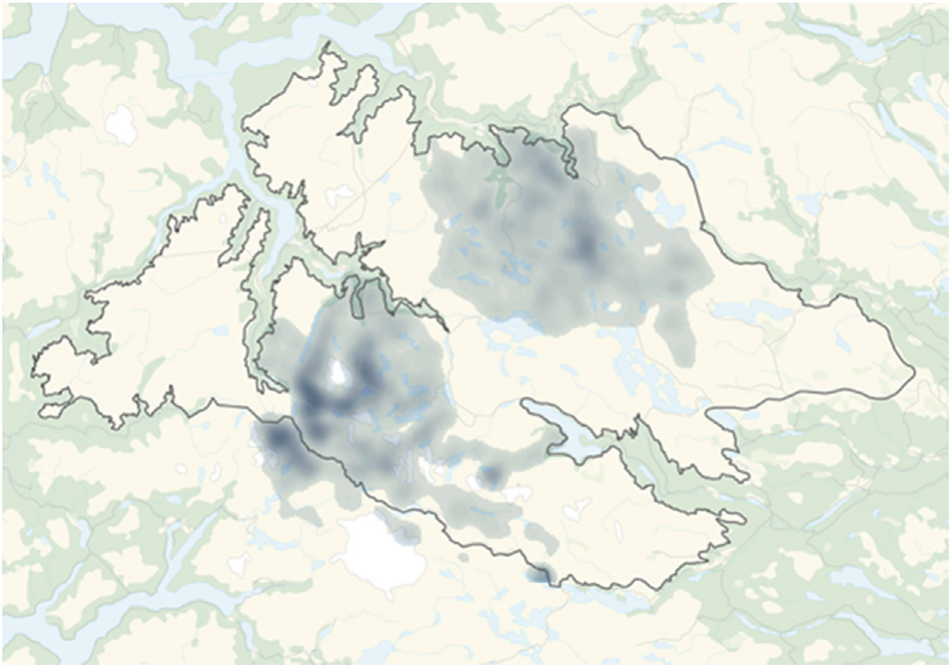
Example of Kernel Density Analysis, visualizing the main grazing area of radio-collared females in the wild reindeer population in Nordfjella during June, July and August. The darker the color, the larger number of GPS positions recorded. The outer limits of the area are marked with a dark line. (For interpretation of the references to color in this figure legend, the reader is referred to the Web version of this article.)

The wild tundra reindeer of southern Norway represent the last naïve remains of this species in Western Europe. Today they are distributed between 23 separate populations, totaling 25,000 to 30,000 wintering animals (WA).This study includes six of these populations (Fig. 1): 1 Forollhogna (2000 WA), 2 Snøhetta (2700 WA), 6 Rondane (3500 WA), 14 Nordfjella (2100 WA), 19 Setesdal Ryfylke (3500 WA) and 20 Setesdal Austhei (2000 WA) (Punsvik and Frøstrup, 2016). The Rondane population is divided into northern and southern subpopulations and this study concerned the southern subpopulation. During the last decade, the positions of each flock, with the exception of Forollhogna, have been monitored by means of GPS-tracked animals. For Forollhogna, standardized field position data is available (from 2015) based on a systematic “reindeer seen” (Sett-rein) registration system. GPS position data for June–August was used to calculate altitude in meters above sea level (m.a.s.l.) of the main summer grazing area of each reindeer population. The number of registered GPS-positions varied between 30,470 and 142,560 per population. For Forollhogna, the calculation was based upon 1009 recorded field positions. ArcMap from ESRI and Spatial Analyst/Kernel Density were used to create “heat maps"/raster maps that visualized the main summer grazing area. We chose to define these maps with 30 classes and made the layer that indicated the least use transparent (Fig. 2). To calculate average altitudes, we ran analyses of the Point Data Sets (GPS and “reindeer seen”) against the Digital Terrain Model from the National Mapping Authority/GeoNorge. “Spatial Analyst/Extract values two points” was used to calculate m.a.s.l. for each position. The exported data set automatically gave us average m.a.s.l. of the main grazing area with standard deviation (SD), and min and max positions.

Wild red deer are widely distributed in southern Norway with the highest densities in the western coastal municipalities. This study encompassed two of these municipalities: 24 Ørsta and 25 Kvinnherad (Fig. 1). GPS position data for June–August was used to calculate altitude in m.a.s.l. of the main summer grazing area. For the Kvinnherad population, position data (33,076 positions) was available from GPS-tracked adult animals in the period 2009–2012. For the Ørsta population, no radio-collar studies have been carried out and therefore position data (51,193 positions) from GPS-tracked adults in three neighboring municipalities Stranda, Norddal and Sykkylven (2014–2015) was used. The m.a.s.l. of each GPS position was calculated using the GIS program QGIS (https://www.qgis.org/en/site/) and a solid digital terrain model with a resolution of 10 × 10 m (Norwegian Mapping Authority, 2017). The altitude information that is the basis of the digital terrain model is primarily height curves and height points from the common map database (FKB). The average m.a.s.l. of the main summer grazing area (June–August) with SD, and min and max positions were calculated.

### 2.2 Climatology

For climatological calculations, a newly developed gridded data set for Norway (seNorge2) was used (Lussana et al., 2018a and b). This is based upon long-term data from all weather stations and makes possible extrapolation of homogenous daily air temperature (2 m above the ground) and precipitation (at 1 × 1 km) for the whole country. The method is particularly robust in the summer when the bias and uncertainties are lower than in winter.

The calculated average elevation of the main summer grazing area, and recorded min and max heights for individual populations were used in the model. Data sets for each population were created from grid cells for the calculated heights (±10 m), centered in each of the areas. The mean summer (June–August) air temperature and monthly precipitation at the average main grazing area and minimum pasture heights were calculated for the last five summers prior to sampling. Long term calculations for the last 60 years (1959–2018) were also carried out.

### 2.3 Hunting and sampling

Sampling was performed from 2012 to 2014 between August 20-September 30 (median August 31) for reindeer and September 1-December 3 (median September 23) for red deer. Faecal samples were collected from the rectum by licensed hunters and sent by mail to the laboratory as soon as possible after hunting. Each sample was accompanied by data on the sex, age (calf, yearling [1.5 yr], or adult [≥ 2.5 yr]), date of hunting and geographical location. Samples lacking age data or with insufficient quantities of faeces were excluded from the study. The number of animals, age distribution and year of sampling for individual populations are shown in Table 1.

**Table 1:** Year and number of calves, yearlings and adults sampled from six wild reindeer (No. 1, 2, 6, 14, 19, 20) and two wild reindeer (No. 24, 25) populations in southern Norway, examined for faecal excretion of first-stage *Elaphostrongylus* and *Dictyocaulus* larvae.

Population	Year	Calves	Yearl	Adults
1 Forollhogna	2012	13	8	26
2 Snøhetta	2013	7	3	20
6 Rondane	2012	10	6	20
14 Nordfjella	2012	4	7	23
19 Setesdal-Ryfylke	2013	11	4	28
20 Setesdal-Austhei	2013	7	4	17
24 Ørsta	2014	21	14	14
25 Kvinnherad	2014	8	18	29
In total		81	64	177

### 2.4 Parasitology

Faecal samples were examined for L1 larvae per gram (LPG; intensity of infection) of *Dictyocaulus* and *Elaphostrongylus*, following a slightly modified Baermann technique (Taylor et al., 2007). Briefly, 10 g of faeces was weighed and placed in a double gauze cloth, closed with a plastic strip and suspended in a conical glass containing lukewarm water overnight. The following day, the faecal parcel was removed and the supernatant aspirated until 5–10 mL liquid remained undisturbed at the bottom the glass. This fluid was drawn off into a 15 mL conical centrifuge tube, the glass rinsed, and this fluid also added to the tube. This was then centrifuged at 1500 rpm for 3 min and the supernatant aspirated, leaving 1 mL undisturbed at the bottom of the tube. A 100 μL subsample was removed from the re-suspended contents of the tube and examined under the microscope at x40-x100magnification. Larvae were identified based on larval morphology and the LPG calculated. In cases in which no larvae were detected in the first subsample, a second 100 μL subsample was examined from the base of the tube after sedimentation had been allowed to occur. If no larvae were observed in this second sample, the result was recorded as no larvae detected. L1 of *E. rangiferi* and *E.cervi* were identified by measuring body length (ten larvae per animal) and the presence of a dorsal spine on the tail (Lankester and Northcott, 1979; Mason, 1995). No other protostrongylid nematodes are known to parasitize reindeer in Europe. The protostrongylid lung worm *Varestrongylus sagittatus* of red deer produces dorsal-spined L1 that can be separated from those of *E. cervi* on the basis of their shorter body length (Mason, 1995). *Dictyocaulus* L1 were identified on shape, length and the presence of dark brown (chromatin) food granules within their intestinal cells (Taylor et al., 2007).

### 2.5 Statistical analyses

The prevalence and intensity data for *Elaphostrongylus* and *Dictyocaulus* larvae were analyzed by a multiple regression analysis, using the R-package *pscl* (Jackman, 2017) and a hurdle model (Zeileis et al., 2008). In a hurdle model, the zero-data are modelled by logistic regression, while counts larger than zero are modelled by a truncated count distribution. Here, we used a truncated negative binomial distribution. The most parsimonious model was selected according to the smallest AICc-value, and we considered an absolute difference in AICc-values less than −2 to be insignificant (Burnham and Anderson, 2002).The full model included age-class, sex and geographical area. Alternative models replacing geographical area with altitude for each main summer grazing area (mean and standard deviation) were also tested. Mean temperature and precipitation for the last five years prior to sampling were not included in the models due to the high correlations between the climatic variables and altitude (see section 3.3). Data from reindeer and red deer were analyzed both separately and together. Analyses were performed in R, version 3.5.2 (R Core Team, 2018).

## 3 Results

### 3.1 Summer pasture altitude

**Fig. 3:**
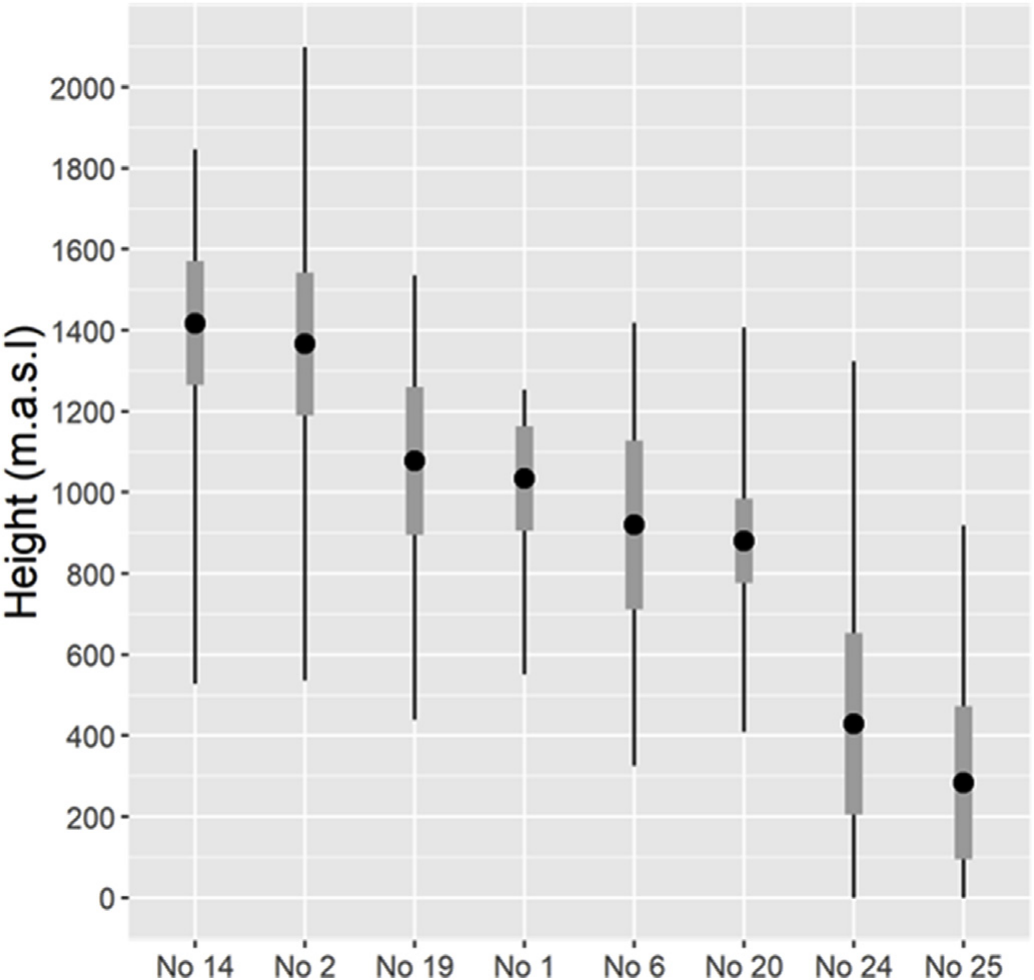
Calculated average altitude above sea level (points) with standard deviation (thicker grey line) of the main summer pasture area of six wild reindeer and two wild red deer populations in South Norway sampled for parasitological studies 2012–2014. The calculation was based upon GPS-positions recorded during June, July and August. The recorded min and max altitudes are indicated by the ends of the black line. Reindeer populations (No): 14 Nordfjella, 2 Snøhetta, 19 Setesdal Ryfylke, 1 Forollhogna, 6 Rondane, 20 Setesdal Austhei. Red deer populations (No): 24 Ørsta, 25 Kvinnherad.

The calculated average altitude of the main grazing area for each population is illustrated in Fig. 3. The grazing areas were significantly higher in Snøhetta (No. 2) and Nordfjella (No. 14), compared to the other four reindeer populations (No. 1, 6, 19, 20). Grazing areas for the two red deer populations were significantly lower than for the reindeer populations with the lowest being Kvinnherad (No. 25).

### 3.2 Parasitological findings

**Fig. 4:**
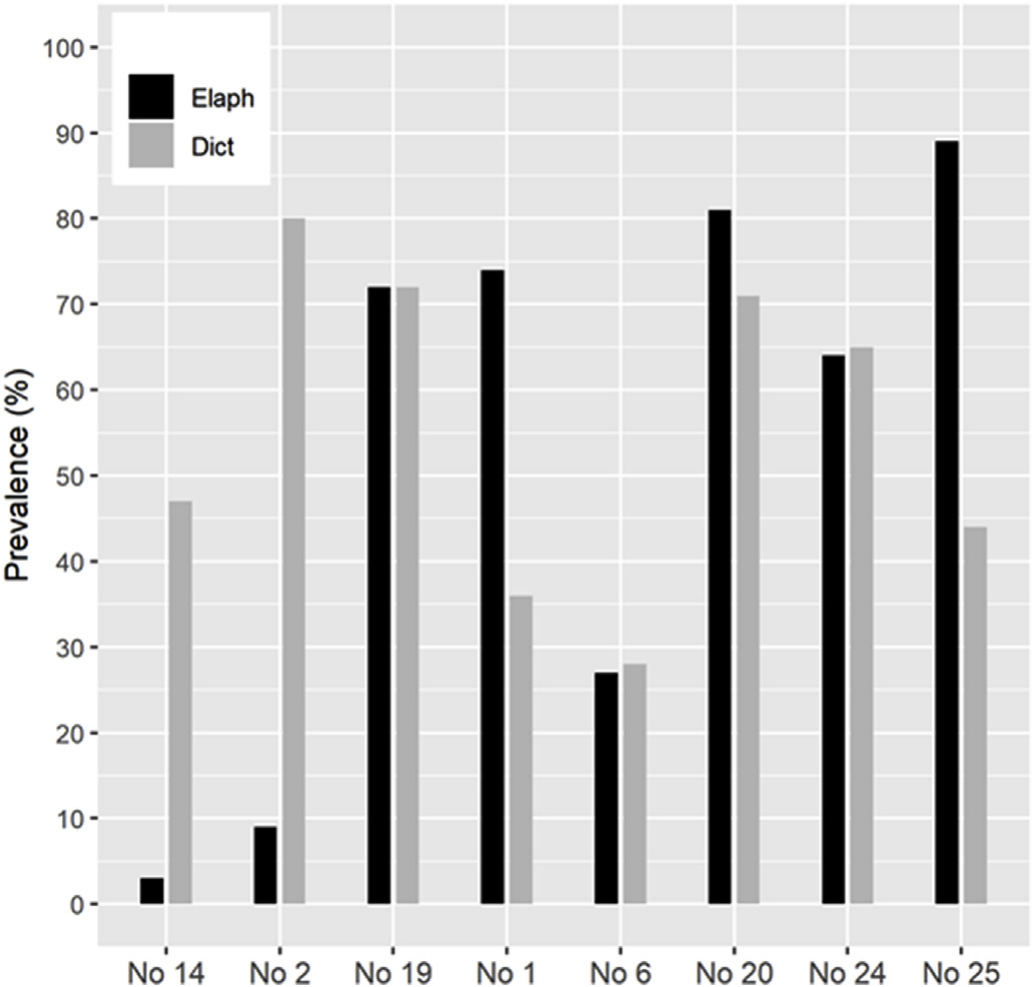
Overall prevalence of *Elaphostrongylus* infection in yearlings and adults (black columns) and *Dictyocaulus* (grey columns) infection in calves, yearlings and adults in six wild reindeer and two wild red deer populations in South Norway. The results are based upon fecal larval detection (patent infection) in animals sampled during hunting in 2012–2014. Calves infected by *Elaphostrongylus* normally are in the pre-patent stage during hunting and were excluded from the graph. Reindeer populations (No): 14 Nordfjella, 2 Snøhetta, 19 Setesdal Ryfylke, 1 Forollhogna, 6 Rondane, 20 Setesdal Austhei. Red deer populations (No): 24 Ørsta, 25 Kvinnherad.

The prevalence of *Elaphostrongylus* and *Dictyocaulus* infections in individual populations are shown in Fig. 4. The average prevalence of *Elaphostrongylus* infection (yearlings, adults) for each species was 46% (76/166) in reindeer and 80% (60/75) in red deer. The average prevalence of *Dictyocaulus* infection (calves, yearlings, adults) was 54% both in reindeer (118/218) and red deer (56/104).

#### 3.2.1 Elaphostrongylus

For reindeer, a significantly lower prevalence of infection was found in the two highest (No. 2, 14) compared to the four lowest (No. 1, 6, 19, 20) located areas (p < 0.0001). When modelling the prevalence by average altitude of the populations’ main summer pasture area, instead of using geographical area as a factor, the model performed equally well according to the AICc-criteria (ΔAICc = 0.5). The general prevalence was significantly higher (p < 0.001) in reindeer adults (68/134; 51%), compared to yearlings (7/32; 22%). This was in contrast to red deer in which the prevalence tended to be higher (p = 0.08) in yearlings (28/32; 88%) than adults (32/43; 74%). Only one red deer calf and one reindeer calf, both killed in September, shed *Elaphostrongylus* L1 larvae. Two red deer from Ørsta shed small numbers of L1 morphologically compatible with *Varestrongylus sagittatus*.

The general intensity of *Elaphostrongylus* infection in reindeer was low, with median and maximum LPGs of 6 and 77, respectively. The general intensity of *Elaphostrongylus* infection was significantly higher in red deer compared to reindeer (p < 0.001), with median and maximum LPGs of 28 and 820. Significantly higher LPGs were found in adult red deer males (p < 0.001), compared to adult females and yearlings.

#### 3.2.2 Dictyocaulus

*Dictyocaulus* spp. was common in all populations and age groups. The general prevalence and intensity of infection did not differ significantly between reindeer and red deer. There was no correlation between prevalence of infection and elevation of the populations’ main summer pasture areas. The median LPG was ≥2 and ≤ 40 for all populations and age groups, except for three adult reindeer and one adult red deer that shed >100 LPG. In reindeer, the general prevalence was lower in calves and female adults compared to male adults and yearlings (p < 0.001). Intensity of *Dictyocaulus* infection in reindeer was higher in adults compared to calves (p = 0.001). In red deer, there were no significant differences in *Dictyocaulus* infection between age groups or sexes.

### 3.3 Short and long-term meteorological data

**Fig. 5:**
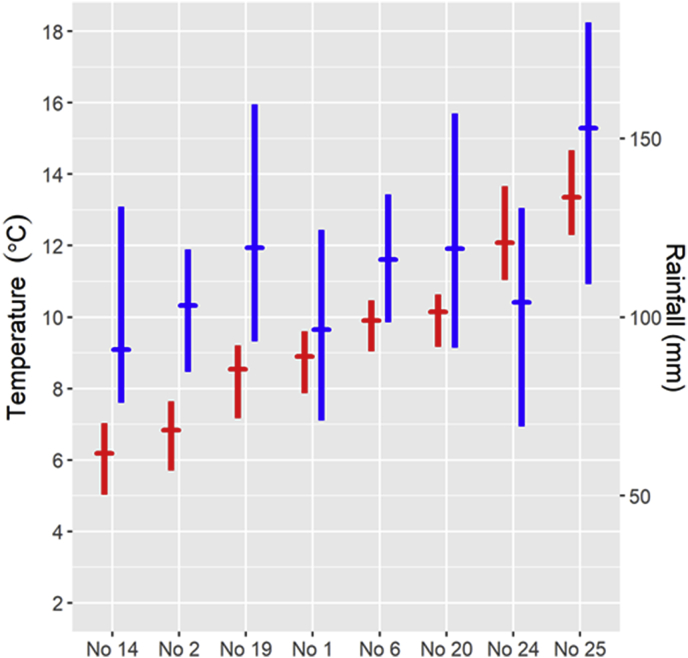
Calculated mean temperature (°C) and monthly rainfall (mm) for June–August at the average altitude of the main summer pasture areas for eight wild reindeer and red deer populations sampled for parasitological studies 2012–2014. The data represent the five summers prior to sampling. The range and average (horizontal bar) of mean monthly temperature (left y-axis) and rainfall (right y-axis) are shown by red and blue lines respectively. Reindeer populations (No): 14 Nordfjella, 2 Snøhetta, 19 Setesdal Ryfylke, 1 Forollhogna, 6 Rondane, 20 Setesdal Austhei. Red deer populations (No): 24 Ørsta, 25 Kvinnherad. (For interpretation of the references to color in this figure legend, the reader is referred to the Web version of this article.)

The calculated mean and range of summer temperature and monthly rainfall for each population's main pasture during the five summers prior to sampling, are illustrated in Fig. 5. There was a negative linear correlation between summer temperature and pasture altitude (Pearson correlation r = −0.993, p < 0.001). Monthly summer precipitation was also (although more weakly) correlated to elevation (r = −0.684, p = 0.06).

**Fig. 6:**
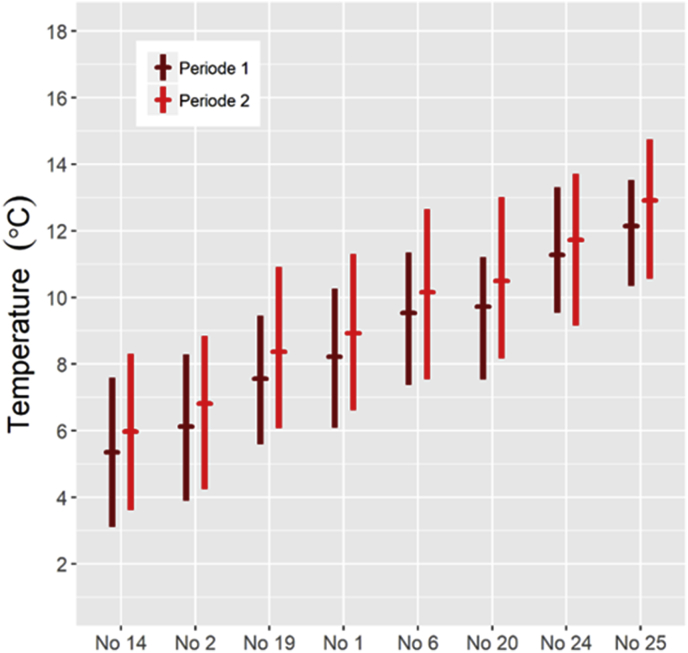
Calculated range and mean (horizontal bar) of temperature for June–August 1959–2018, at the average altitude of the main summer pasture areas for eight wild reindeer and red deer populations sampled for parasitological studies 2012–2014. The results are presented separately for the two 30-year periods 1959–1988 (brown symbols) and 1989–2018 (red symbols). Reindeer populations (No): 14 Nordfjella, 2 Snøhetta, 19 Setesdal Ryfylke, 1 Forollhogna, 6 Rondane, 20 Setesdal Austhei. Red deer populations (No): 24 Ørsta, 25 Kvinnherad. (For interpretation of the references to color in this figure legend, the reader is referred to the Web version of this article.)

The calculated mean and range of summer temperature for each population's main pasture during the periods 1959–1988 and 1989–2018, are illustrated in Fig. 6. For all areas, there was a significant increase in the mean summer temperature of 0.5–0.8 °C for individual populations (average: 0.7 °C) between the two 30-year-periods (p < 0.001). Only a tendency towards increased rainfall was observed (p = 0.09) from the first to the second period.

**Fig. 7:**
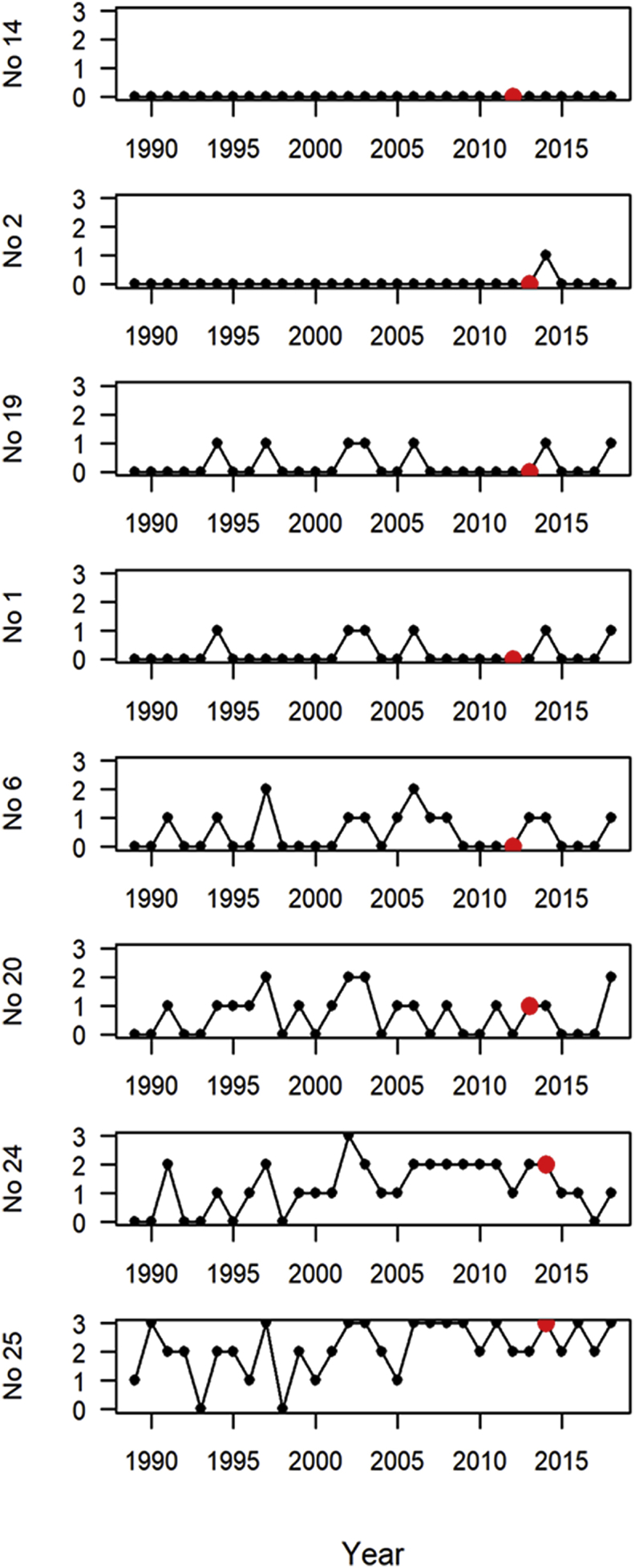
Annual number of summer months (June–August) 1989–2018 with a calculated mean temperature ≥12 °C in the main summer grazing area of six wild reindeer populations and two red deer municipalities in South Norway sampled for parasitology 2012–2014. The sampling year of each population is indicated by a red dot. Reindeer populations (No): 14 Nordfjella, 2 Snøhetta, 19 Setesdal Ryfylke, 1 Forollhogna, 6 Rondane, 20 Setesdal Austhei. Red deer populations (No): 24 Ørsta, 25 Kvinnherad. (For interpretation of the references to color in this figure legend, the reader is referred to the Web version of this article.)

The annual number of months in each main summer pasture area with a mean temperature ≥12 °C for the period 1989–2018 is shown in Fig. 7. A temperature of ≥12 °C for at least two months was reached for all populations in most years at their lowest recorded summer positions.

## 4 Discussion

The present study demonstrated a marked reduction in prevalence and intensity of *Elaphostrongylus* infection with increasing altitude and reduced temperature in the main summer grazing areas. We consider these findings to be closely related to the temperature-dependent development of L1 larvae to infective L3 larvae in gastropods (Halvorsen and Skorping, 1982). Whether differences in gastropod density between the areas could also be a contributing factor is not known (gastropods are common in Norwegian mountain areas). According to our calculations, a sufficiently high temperature is not maintained over a period long enough to allow development of L3 in the course of a single summer in the main summer pasture areas of the two highest located reindeer populations. While occurring only exceptionally in the other reindeer areas, these conditions are met regularly in red deer areas. It may therefore be speculated that while red deer may become infected over their entire main summer grazing areas, reindeer infection mainly occurs at relatively low altitude, in areas with restricted animal residence time and possibility of infection. Halvorsen and Skorping (1982) have hypothesized that the parasite may compensate for low temperature by fulfilling its larval development in overwintering gastropods. This could be a factor contributing to maintenance of infection in reindeer pastures, especially in the two highest located populations. The patent infection identified in single reindeer and red deer calves may reflect ingestion of L3-containing gastropods in early summer, in which larval development had started the previous year.

A higher *Elaphostrongylus* infection pressure in red deer compared to reindeer was reflected both through a generally higher intensity of infection and a larger proportion of yearlings infected (reflecting infection as calves) in the red deer populations. With reference to earlier studies (Prosl and Kutzer, 1982; Halvorsen et al., 1985), we consider that the intensity of infection identified was low in reindeer and moderate in red deer. The higher intensity of infection found in adult male red deer, compared to adult females and yearlings could be a result of increased larval production due to stress-induced immune suppression during the autumn rutting season in adult males (Gaudernack et al., 1984; Halvorsen et al., 1985). For reindeer, the prevalence of infection found in yearlings was less than half of that found in adults. This was in contrast to red deer in which the prevalence was similar in yearlings and adults. These findings indicate a time of infection as calves, yearlings or young adults in the reindeer, and as calves in red deer. Consequently, clinical elaphostrongylosis should be a considered diagnosis among calves and young animals in reindeer and among calves in red deer. However, our data suggest mild to moderate infections, with presumably few clinical effects at the population level. Nevertheless, it should be emphasized that even slight clinical signs occurring in late autumn and winter may be critical, especially in the harsh environment inhabited by wild reindeer.

An increasing level of clinical significance of *Elaphostrongylus* infection can be expected in light of climate warming, as illustrated by the 0.7 °C increase in mean temperature from the period 1959–1988 to 1989–2018 found in the present study. This increase in clinical significance could be especially valid for reindeer, as suggested by Handeland and Slettbakk (1994). The authors speculated that, due to the marginal summer temperatures in reindeer areas, the animals may have developed little specific immunity prior to heavy infections that may occur within a restricted period of time, following hot summers. This was considered in contrast to cervids (red deer) living in temperate areas that, due to more favorable condition for development of L3 in gastropods, probably ingest L3 over a longer summer period, and thus develop more immunological protection prior to the main season of infection in late summer.

*Dictyocaulus* infection was common in all populations and age groups of reindeer and red deer and precipitation levels seem adequate for larval development in all studied environments. The highest prevalence and intensity of *Dictyocaulus* infection was found in the reindeer population in Snøhetta (Fig. 4 - No. 2, intensity data not shown). *Dictyocaulus* infection (*D. eckerti*) is also prevalent in the Norwegian muskox population located in the Snøhetta area (Davidson et al., 2014). The mechanism of transmission of *Dictyocaulus* spp. from one year to another is through overwintering larvae in the pasture, or hypobiotic larvae in the lungs of carrier animals (Taylor et al., 2007). A low freeze tolerance has been demonstrated for larvae of *D. viviparus* (Rose, 1956) whereas the freeze tolerance for *Dictyocaulus* spp. in cervids is unknown. However, the winter conditions in the summer grazing areas of the populations examined in this study are harsh, especially for reindeer and we consider overwintering as hypobiotic larvae in the lungs to be the most likely way of inter-annual transmission.

The interpretation of intensity of *Dictyocaulus* infection based on LPG is challenging since it may vary throughout summer (Prosl and Kutzer, 1982; Davidson et al., 2014) and due to a lack of good reference values. For *D. filaria* infection in small ruminants, levels between 2 and 100 LPG are considered to reflect moderate and >100 LPG high degrees of infection respectively (DTU Veterinærinstituttet, 2009). By these standards, the infections identified in the present study were of moderate and only exceptionally high grade. A high clinical significance of *Dictyocaulus* infection and death among calves has been reported in farmed deer in various countries (Charleston, 1980; Mason, 1995; Corrigall et al., 1980). Harsh climatic conditions, poor pasture and poor body condition have been suggested as contributing factors (Corrigall et al., 1980). The significance of dictyocaulosis in wild cervid populations is poorly documented. However, several reports suggest this infection to be a common cause of death in young wild red deer (Dunn, 1967) and reindeer (Rahko et al., 1992) in Europe, and in caribou and muskox in North America (see Kutz et al., 2012). In reindeer herds in northern Norway, outbreaks of dictyocaulosis with or without contribution of opportunistic bacteria (*Pasteurella multocida*), have caused severe losses of calves in late April and early May (Kummeneje, 1977). These outbreaks were presumably caused by hypobiotic larvae, maturing in the lungs in spring. Dictyocaulosis in deer is characterized by reduced food intake and rapid loss of body condition without typical respiratory signs and may be challenging to recognize in live animals (Corrigall et al., 1980). Based on our findings, we suggest *Dictyocaulus* infection to be an unrecognized cause of disease in our wild reindeer and red deer populations.

In conclusion, this study demonstrated that *Elaphostrongylus* and *Dictyocaulus* infections are endemic and highly prevalent in Norwegian wild reindeer and red deer populations. We consider *Dictyocaulus* infection to be a potential cause of disease and death in both species, especially among poorly nourished calves in spring. For reindeer, we predict an increased significance of *Elaphostrongylus* infection in the face of climate warming.

## Declarations of interest

None.

## References

[bib1] Bakka K., Haukalid S., Sætre E.M. (2006). Endoparasites in Red Deer (In Norwegian with English Abstract).

[bib2] Bischof R., Loe L.E., Meisingset E.L., Zimmermann B., Van Moorter B., Mysterud A. (2012). A migratory northern ungulate in the pursuit of spring: jumping or surfing the green wave. Am. Nat..

[bib3] Burnham K.P., Anderson D.R. (2002). Model Selection and Multimodel Inference: A Practical Information-Theoretic Approach.

[bib4] Charleston W.A.G. (1980). Lungworm and lice of the red deer (*Cervus elaphus*) and fallow deer (*Dama dama*) – a review. N. Z. Vet. J..

[bib5] Corrigall W., Easton J.F., Hamilton W.J. (1980). *Dictyocaulus* infection in farmed red deer (*Cervus elaphus*). Vet. Rec..

[bib6] Davidson R.K., Amundsen H., Oftenes Lie N., Luyckx K., Robertson L.J., Verocai G.G., Kutz S.J., Ytrehus B. (2014). Sentinels in a climatic outpost: endoparasites in the introduced muskox (*Ovibus moschatus wardi*) population of Dovrefjell, Norway. Int. J. Parasitol. Parasites Wildl..

[bib7] Dunn A.M. (1967). Endoparasites of deer. Deer.

[bib8] Fletcher T.J. (1982). Management problems and disease in farmed deer. Vet. Rec..

[bib9] Gaudernack G., Halvorsen O., Skorping A., Stokkan K.A. (1984). Humural immunity and output of first stage larvae of *Elaphostrongylus rangiferi* (Nematoda, Metastrongyloidea) by infected reindeer, *Rangifer tarandus tarandus*. J. Helminthol..

[bib10] Gibbons L.M., Halvorsen O., Stuve G. (1991). Revision of the genus *Elaphostrongylus* Cameron (Nematoda, Metastrongyloidea) with particular reference to species of the genus occurring in Norwegian cervids. Zool. Scr..

[bib11] Halvorsen O., Skorping A. (1982). The influence of temperature on growth and development of the nematode *Elaphostrongylus rangifer*i in the gastropods *Arianta arbustorum and Euconulus fulvus*. Oikos.

[bib12] Halvorsen O., Andersen J., Skorping A., Lorentzen G. (1980). Infection in reindeer with the nematode *Elaphostrongylus rangiferi* MITSKEVICH in relation to climate and distribution of intermediate hosts. Proceedings of the 2nd International Reindeer/Caribou Symposium DVF.

[bib13] Halvorsen O., Skorping A., Hansen K. (1985). Seasonal cycles in the output of first stage larvae of the nematode *Elaphostrongylus rangiferi* from reindeer, *Rangifer tarandus tarandus*. Polar Biol..

[bib14] Handeland K. (1994). Experimental studies of *Elaphostrongylus rangiferi* in reindeer (*Rangifer tarandus tarandus*): life cycle, pathogenesis, and pathology. J. Vet. Med. B.

[bib15] Handeland K., Slettbakk T. (1994). Outbreaks of clinical cerebrospinal elaphostrongylosis in reindeer (*Rangifer tarandus tarandus*) in Finnmark, Norway, and their relation to climatic conditions. J. Vet. Med. B.

[bib16] Handeland K., Skorping A., Stuen S., Slettbakk T. (1994). Experimental studies of *Elaphostrongylus rangiferi* in reindeer (*Rangifer tarandus tarandus*): clinical observations. Rangifer.

[bib17] Handeland K., Gibbons L.M., Skorping A. (2000). Aspects of the life cycle and pathogenesis of *Elaphostrongylus cervi* in red deer (*Cervus elaphus*). J. Parasitol..

[bib18] Hemmingsen W., Halvorsen O., Skorping A. (1993). Migration of adult *Elaphostrongylus rangiferi* (Nematoda: protostrongylidae) from the spinal subdural space to the muscles of reindeer (*Rangifer tarandus*). J. Parasitol..

[bib19] Jackman S. (2017). Pscl: Classes and Methods for R Developed in the Political Science Computational Laboratory. https://github.com/atahk/pscl.

[bib20] Kummeneje K. (1977). *Dictyocaulus viviparus* infestation in reindeer in northern Norway. Acta Vet. Scand..

[bib21] Kutz S.J., Ducrocq J., Verocai G.G., Hoar B.M., Colwell D.D., Beckmen K.B., Polley L., Elkin B.T., Hoberg E.P. (2012). Parasites of ungulates of Arctic North America and Greenland: a view of contemporary diversity, ecology, and impact in a world under change. Adv. Parasitol..

[bib22] Lankester M.W., Northcott T.H. (1979). *Elaphostrongylus cervi* CAMERON, 1931 (nematoda: metastrongyloidea) in caribou (*Rangifer tarandus caribou*) of newfoundland. Can. J. Zool..

[bib23] Lussana C., Saloranta T., Skaugen T., Magnusson J., Tveito O.E., Andersen J. (2018). seNorge2 daily precipitation, an observational gridded dataset over Norway from 1957 to the present day. Earth Syst. Sci. Data.

[bib24] Lussana C., Tveito O.E., Uboldi F. (2018). Three-dimensional spatial interpolation of 2 m temperature over Norway. Q. J. R. Meteorol. Soc..

[bib25] Mason P. (1995). *Elaphostrongylus cervi* and its close relatives; a review of protostrongylids (Nematoda, Metastrongyloidea) with spiny-tailed larvae. Surveillance.

[bib26] Mitskevich V.Y., Boev S.N. (1964). Life cycle of Elaphostrongylus rangiferi MIZ.

[bib27] Mysterud A., Loe L.E., Zimmermann B., Bischof R., Veiberg V., Meisingset E. (2011). Partial migration in expanding red deer populations at northern latitudes – a role for density dependence?. Oikos.

[bib28] Ottestad A.K. (1983). The occurrence of *Elaphostrongylus cervi* in a population of red deer (*Cervus elaphus*). (In Norwegian with English Summary).

[bib29] Panin V.Y., Boev S.N. (1964). Tsikl razvitiya Elaphostrongylus panticola lubimov, 1945 (life cycle of Elaphostrongylus panticola lubimov, 1945). Parazity Se'lskokhozyaistvennykh Kazakhstana, 3 (Parasites of Farm Animals in Kazakhstan.

[bib30] Prosl H., Kutzer E. (1980). Zur Pathologie des Elaphostrongylusbefalles beim Rothirsch (*Cervus elaphus hippelaphus*) (In German with English summary). Monatsh. Veterinärmed..

[bib31] Prosl H., Kutzer E. (1982). Jahresrhythmus in der Larvenausheidung von *Dictyocaulus viviparus, Varestrongylus sagittatus* and *Elaphostrongylus cervi* bei Rotwild (*Cervus elaphus*). (In German with English summary). Angew. Parasitol..

[bib32] Punsvik T., Frøstrup J.C. (2016). The Wild Reindeer: Biology-History-Management (In Norwegian).

[bib33] Pyziel A.M., Laskowski Z., Demiaszkiewics A.W., Höglund J. (2017). Interrelationships of *Dictyocaulus* spp. in wild ruminants with morphological description of *Dictyocaulus cervi* n. sp. (nematode: trichostrongyloidea) from red deer, *Cervus elaphus*. J. Parasitol..

[bib34] R Core Team (2018). R: A Language and Environment for Statistical Computing. https://www.R-project.org/.

[bib35] Rahko T., Saari S., Nikander S. (1992). Histopathological lesions in spontaneous dictyocaulotic pneumonia of reindeer (*Rangifer tarandus tarandus*). Rangifer.

[bib36] Rose J.H. (1956). The bionomics of the free-living larvae of *Dictyocaulus viviparous*. J. Comp. Pathol..

[bib37] Skorping A., Halvorsen O. (1980). The susceptibility of terrestrial gastropods to experimental infection with *Elaphostrongylus rangiferi* MITSKEVICH (Nematoda: metastrongyloidea). Z. Parasitenkd..

[bib38] Taylor M.A., Coop R.L., Wall R.L. (2007). Veterinary Parasitology.

[bib39] Veterinærinstituttet D.T.U. (2009). Parasites in sheep and goats (in Danish: parasitter hos får og ged). User Manual 2009 (Brugerhåndbog 2009).

[bib40] Zeileis A., Kleiber C., Jackman S. (2008). Regression models for count data in R. J. Stat. Softw..

